# Effect of *Spirulina Nigrita*^®^ Supplementation on Indices of Exercise-Induced Muscle Damage after Eccentric Protocol of Upper Limbs in Apparently Healthy Volunteers

**DOI:** 10.3390/nu16111651

**Published:** 2024-05-28

**Authors:** Anastasios Krokidas, Athanasios G. Gakis, Ourania Aktypi, Smaragdi Antonopoulou, Tzortzis Nomikos

**Affiliations:** Department of Nutrition and Dietetics, School of Health Science and Education, Harokopio University of Athens, 176 76 Athens, Greece; akroki@hua.gr (A.K.); thgakis@hua.gr (A.G.G.); oaktipi@gmail.com (O.A.); antonop@hua.gr (S.A.)

**Keywords:** exercise, eccentric, muscle damage, inflammation

## Abstract

Spirulina is a supplement with antioxidant and anti-inflammatory properties that may enhance performance and recovery after intense exercise. The present study aimed to investigate the effects of *Spirulina Nigrita*^®^ on physical performance, and recovery markers after intense eccentric exercise in healthy moderately physically active volunteers. In a double-blind crossover design, participants were supplemented either with spirulina (42 mg Kg^−1^ BW per day) or a placebo for 15 days before conducting an eccentric exercise protocol using the non-dominant arm. A six-week washout period was required between conditions. Performance and mobility markers such as isometric peak torque (PTQ), ligament range of motion (ROM), and perceived muscle discomfort (VAS) were assessed and blood samples (CK, LDH) were obtained at 1, 24, 48, and 72 h post-exercise. No significant differences were noticed between the two conditions on any of the investigated markers, indicating that spirulina supplementation has no positive effect on isometric muscle performance or alleviation of exercise-induced muscle damage (EIMD) symptoms in the specific population.

## 1. Introduction

Exercise-induced muscle damage (EIMD) is a complex physiological phenomenon that occurs mainly because of intense or unaccustomed eccentric exercise or unaccustomed training. It is characterized by structural and functional alterations in skeletal muscle fibers, leading to mechanical and metabolic stress within the fibers that are activated during the exercise stimulus [1,2]. Its clinical symptoms include muscle soreness, swelling, reduced strength, and impaired range of motion [3,4]. The underlying mechanisms involve mechanical stress, oxidative stress, inflammation, and disruption of the excitation–contraction coupling process [5]. Recent studies have shed light on the potential role of specific signaling pathways, including nuclear factor-kappa B (NF-κB), mitogen-activated protein kinases (MAPKs), and reactive oxygen species (ROS), in the development of EIMD [3]. Understanding these mechanisms and their multifaceted nature is crucial for optimizing exercise regimens, preventing injuries, and promoting effective recovery strategies.

In addition, eccentric exercise-induced muscle damage seems to be a useful inflammatory model, which can be applied to humans for the investigation of the mechanisms regulating the response to inflammatory stimuli. It is also a useful model for the assessment of the anti-inflammatory properties of foods, supplements, and dietary patterns in dietary intervention studies and clinical trials with human subjects [6]. Eccentric exercise is characterized by acute inflammatory responses within the damaged muscle fibers, leading to an influx of immune cells and the release of various inflammatory mediators [7]. This model provides a controlled and reproductive way to investigate the cellular and molecular mechanisms that lead to inflammation and its implications for muscle repair and adaptation [8,9]. Research utilizing this model has demonstrated increased expression of pro-inflammatory cytokines, such as interleukin-6 (IL-6) and tumor necrosis factor-alpha (TNF-α), as well as activation of nuclear factor-kappa B (NF-κB), a transcription factor involved in inflammatory gene regulation [5,7]. Moreover, recent studies have shed light on the resolution phase of inflammation, highlighting the involvement of anti-inflammatory cytokines, such as interleukin-10 (IL-10), and the importance of a timely resolution for optimal muscle regeneration [9,10]. These findings emphasize the intricate balance between pro-inflammatory and anti-inflammatory processes in the context of EIMD and eccentric exercise, providing insights into the regulation of muscle inflammation and potential therapeutic strategies for enhancing recovery and adaptation [3,11,12].

Spirulina, a blue-green microalgae, has garnered attention due to its potential antioxidative and anti-inflammatory properties. It is rich in bioactive compounds such as phycocyanin, chlorophyll, carotenoids, proteins, vitamins (beta-carotene, vitamin B12, and vitamin E), minerals, and pigments which contribute to its antioxidant capacity [13]. Moreover, spirulina has been shown to exhibit anti-inflammatory properties by modulating inflammatory pathways, increasing the activity of NK cells, and reducing the production of pro-inflammatory cytokines, such as tumor necrosis factor-alpha (TNF-α) and interleukin-6 (IL-6) [14,15]. Furthermore, spirulina improved the lipid profile, immune resistance, and antioxidant capacity in an elderly population [16].

Its anti-inflammatory and antioxidant properties make spirulina a promising candidate for contributing to the reduction of the symptoms of EIMD. The available data in the literature are controversial concerning the effects of spirulina on EIMD and oxidative stress. Indeed, studies have reported positive results, suggesting that spirulina supplementation reduced the concentration of the lipid peroxidation product malondialdehyde (MDA) and activated the antioxidant enzymes superoxide dismutase (SOD) and glutathione peroxidase (GPX) [17]. Similarly, other studies reported that spirulina supplementation attenuated EIMD symptoms, improved exercise performance, enhanced antioxidant capacity, and increased aerobic exercise performance [18,19,20]. On the other hand, there are studies yielding null or inconclusive results concerning EIMD indices [21,22]. Variations in the study design, dosage, duration of supplementation, exercise protocol, and participant characteristics could contribute to these conflicting findings. Furthermore, methodological limitations, such as small sample sizes, inadequate control groups, or lack of dosage standardization may affect the interpretation of results. Further well-designed, randomized controlled trials are necessary to elucidate the true effects of spirulina supplementation on these exercise-related outcomes.

Most of the aforementioned studies used supplements of *Spirulina plantensis* and muscle damage protocols of the lower limbs. In our study, the volunteers were provided with a high-quality *Spirulina Nigrita*^®^ supplement, rich in protein and antioxidants, while the muscle damage protocol of the upper libs was applied. Despite the lower muscle mass of the upper limbs, they are less adapted to eccentric contractions and therefore more prone to EIMD-induced inflammation and oxidative stress [23,24]. Considering that the *Spirulina Nigrita*^®^ strain is a very popular supplement among athletes and physically active populations in Greece with health claims such as antioxidant, and anti-inflammatory, based on no clinical studies at all, we aimed to investigate the effect of *Spirulina Nigrita*^®^ supplementation on clinical, physiological, and biochemical indices of muscle damage induced by eccentric exercise protocol on healthy moderate physically active volunteers. Taking into account that oxidative stress and inflammation underlie the pathophysiology of exercise-induced muscle damage, we hypothesize that due to the putative antioxidant and anti-inflammatory properties, *Spirulina Nigrita*^®^ supplementation will alleviate symptoms of EIMD after eccentric exercise.

## 2. Materials and Methods

### 2.1. Study Design and Baseline Measurements

**Figure 1:**
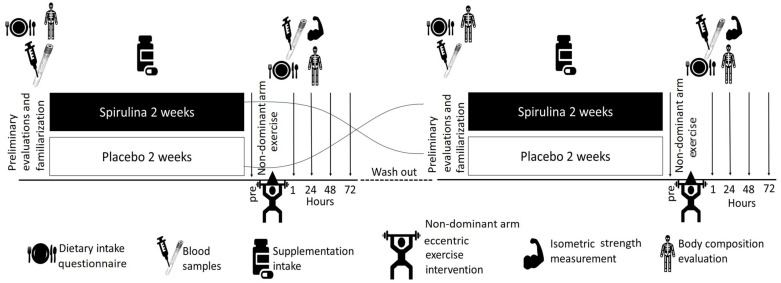
Study design.

A double-blind, placebo-controlled, randomized crossover design was used. The intervention consisted of two arms. In each arm, every volunteer was administered, in random order, either the spirulina supplement (SPI) or placebo (PLA) for 15 days before conducting an eccentric exercise protocol. The randomization was performed with an MS Excel algorithm creating a random sequence of numbers (i.e., 01001…). Participants were allocated to SPI/PLA (if 0) or PLA/SPI (if 1). They were asked to maintain their usual physical activity lifestyle and dietary habits during the entire period of the protocol. Upon completion of the spirulina or placebo intervention period, subjects visited the laboratory for 4 consecutive days. On the first day, a muscle-damaging eccentric exercise protocol was performed. Before the exercise protocol and 1, 24, 48, and 72 h after the completion of the protocol, ergometric measurements, subjective feelings of discomfort, and biological samples were obtained. The two arms were separated by a six-week washout period to avoid the carry-over effect of each supplementation period and the repeated bout effect of eccentric exercise. The experimental protocol was approved by the Ethics Committee of Harokopio University (Reg. No. 2759/16090-2019). The protocol was conducted in compliance with both Good Clinical Practices and the Declaration of Helsinki. All the participants were previously informed about the study objectives, as well as possible risks, discomforts, and benefits, before obtaining their written consent (Figure 1).

### 2.2. Participants

The study included 14 healthy men and women (aged 24.5 ± 3.8 years; BMI 23.8 ± 4; body fat 21.2 ± 5%). Inclusion criteria were as follows: (1) apparently healthy individuals of both genders, aged 18–30, (2) non-smokers, and (3) moderate physical activity profile without regular participation in training sessions (either aerobic or anaerobic). Exclusion criteria included the following: (1) tobacco or smoking product use; (2) intake of any kind of supplement; (3) known allergies to algae; (4) adoption of extreme dietary habits; (5) medical treatment excluding thyroid hormones; and (6) known musculoskeletal injuries or chronic diseases.

### 2.3. Supplementation

The *Spirulina Nigrita*^®^ powder was kindly provided by the manufacturer (*Spirulina Nigrita*^®^, Therma Nigrita, Serres, Greece, www.spirulinanigrita.com, accessed on 15 September 2021) and encapsulated in 500 mg containing capsules by a certified laboratory. Visually identical 500 mg maltodextrin-containing capsules were given as the placebo. According to the body mass, each participant consumed 6–12 capsules per day to achieve a daily intake of ~42 mg∙kg^−1^ body weight. The capsules were taken before meals (one-third of capsules before each meal) for 15 days. All volunteers were asked to bring back the remaining capsules. We assessed compliance by comparing the remaining capsules with those initially given to the volunteers. The compliance for the placebo was 89% and the compliance for spirulina was 93%.

### 2.4. Anthropometry

Base-line measurements were performed 2–3 days before the initiation of the exercise protocol for each period. Body weight was measured to the nearest 0.5 kg, standing height to the nearest 0.5 cm (Seca, Birmingham, UK), and BMI was calculated by the formula Body Weight/[Height]^2^. Waist circumference was measured between the superior iliac crest and the lower rib margin in the midaxillary line to the nearest 0.1 cm. Moreover, body composition analysis was determined using the dual-energy X-ray absorptiometry method (DXA), and body scanners (model DPX, Lunar Corp., Madison, WI, USA, software version 3.6), at moderate speed according to the instructions of the manufacturer. Resting arterial blood pressure and heart rate were measured three times in the left arm in a sitting and resting position by the electronic device “Omron M6 Comfort” (Table 1).

### 2.5. Estimation of Physical Activity

The physical activity profile was estimated with a translated short version of the International Physical Activity Questionnaire (IPAQ). The sum of the weekly multiples of the resting metabolic rate (MET-minutes per week) from walking, moderate- and vigorous-intensity exercise was calculated [25].

### 2.6. Estimation of Dietary Intake

To evaluate the participants’ dietary intake and to ensure that their eating habits did not change between arms, both a baseline recording of eating habits over the past month (Food Frequency Questionnaire (FFQ) [26] and an assessment of dietary intake with 24 h dietary recalls were performed. The FFQ estimated the frequency of the consumption of the main food items and the MedDiet Score. The 24 h dietary recalls were completed the day before the exercise, a random day of the previous week, and a weekend day. The 24 h recall analysis was performed using the USDA database (http://ndb.nal.usda.gov/ accessed on 12 June 2023) and Trihopoulou database for traditional Greek recipes using Nutritionist Pro™ software (version 2.2, Axxya Systems LLC, Stafford, TX, USA) [27]. The intake of macro- and micronutrients per recall was calculated [27] and the mean daily intake was calculated (Table 2). All questionnaires were completed through an interview (face-to-face or by a phone call).

### 2.7. Eccentric Exercise Protocol

Subjects performed a 10 min warm-up of hand-cycling (50–70 rpm) on “jocca hand-cycle”, prior to eccentric exercise protocol, followed by 2 min of dynamic stretching exercises of the major muscle groups of the upper limbs. An eccentric exercise session was performed on an isokinetic dynamometer (BIODEX System 3 Pro, New York, NY, USA), and exercise protocols were undertaken from the seated position (120° hip angle). Participants were positioned on an isokinetic dynamometer and stabilized with an arm curl bench at 45° of shoulder flexion according to the manufacturer’s instructions. The angle ligament aligned with the axis of rotation of the dynamometer. The protocol included 10 sets of 8 repetitions of elbow flexor muscles of the volunteers’ non-dominant arm, as was personally precepted “less skillful” by the volunteer, with 2 min rest between sets. In each eccentric contraction, the range of motion was set at 90° with an initial elbow angle of 20°, a final elbow angle of 110°, and an angular velocity of 30 deg/s. Each repetition was separated by a 6 s rest period. The lever arm was returned to the start position at 30 deg/s via software command with no need to generate any active tension by the subject. The investigator encouraged the participants throughout the exercise to apply maximal resistance against the lever arm of the isokinetic dynamometer.

### 2.8. Measurement of EIMD Indices

For the evaluation of muscle damage, isometric peak and average peak torque of biceps brachii were measured. A 5 min warm-up of hand-cycling (50–70 rpm) and dynamic stretching exercises of the major upper limbs muscle groups were performed, followed by maximum isometric torque production capacity test of the non-dominant brachii flexor muscles on isokinetic dynamometer (BIODEX System 3 Pro, NY, USA). The arm was stabilized with Velcro straps and the center of the elbow ligament was aligned with the axis of rotation of the dynamometer, positioned at 90° according to the manufacturer’s instructions. The participants were verbally motivated to develop maximum strength at the fastest possible rate and try to maintain maximum force levels for 3 s. Each trial was repeated 3 times with 2 min rest between and maximum torque value of the three trials was used [28].

The assessment of delayed-onset muscle soreness was performed using a visual analog scale (VAS), where participants indicated the corresponding point of perceived discomfort experienced on a 100 mm graded scale when the researcher palpated the area.

The joint angle when participants intentionally fully flexed their arm by their side was defined as flexed angle (FANG). The joint angle when they extended the elbow joint as much as possible was defined as the extended elbow joint angle (EANG). Both angles were measured using a goniometer (Gima 27340, Italy). The angle subtracting the FANG from the EANG was deemed to be the elbow joint’s range of motion (ROM) [29]. Additionally, circumference of the humerus at its midpoint was determined using a tape measure at the midpoint of the biceps brachii.

### 2.9. Blood Collection and Isolation of Biological Samples

Blood samples were collected from an antecubital arm vein using a disposable 21-gauge needle. Blood samples were collected immediately before exercise and at 1, 24, 48, and 72 h post-exercise. Serum was isolated from vacutainers with no anticoagulant by centrifugation (1500× *g*, 20 min, 4 °C) after allowing the blood to clot for 15 min at room temperature. Plasma was isolated from EDTA-anticoagulated vacutainers by centrifugation (1500× *g*, 10 min, 4 °C). The blood samples of the female participants were collected during the follicular phase of the menstrual cycle, in which muscle strength is not significantly changed [30]. The time between the end of the first bout of exercise and the start of the second bout of exercise was 8 weeks (washout 6 weeks plus 2 weeks second supplementation period) which allowed us to collect the blood samples at approximately the same phase of the menstrual cycle. Serum and plasma samples were stored at −80 °C until analysis.

### 2.10. Biochemical Analyses

Creatine kinase (CK) and lactate dehydrogenase (LDH) activities were determined by enzymatic photometric methods using commercially available kits (Biosis, Athens, Greece) adjusted for 96-well plates in accordance with the manufacturer’s instructions. The mean intra-individual coefficient of variation for CK and LDH were 22% and 28%, respectively (n = 6).

### 2.11. Statistical Analysis

An *a priori* power analysis was conducted using G-power 3.1 software assuming sphericity and homogeneity of variances across treatments. Using an a priori test for repeated measures ANOVA with a within-subjects design, an ES 0.25, alpha 0.05, power 0.8, 1 group, 10 measures (2 situations × 5 times), correlation of 0.5, and nonsphericity correction of 1, the sample size required was 14. Using these parameters and a sample size of 11, the power was 0.68.

Categorical variables are presented as frequencies. Normally distributed continuous variables are presented as mean ± standard error (SE), whereas non-normally distributed continuous variables are presented as median (25th, 75th percentiles). Kolmogorov-Smirnov and Shapiro–Wilk tests confirmed data normality. Paired-sample *t*-tests or Wilcoxon tests were used to compare the baseline characteristics of the volunteers before each intervention.

Two-way (time [PreEx, PostEx, 24 h, 48 h, 72 h] × condition [PLACEBO, SPIRULINA]) repeated-measures analysis of variance were used for statistical analyses. Sphericity was checked by Mauchly’s test, and Greenhouse–Geisser correction on degrees of freedom was done if necessary. In the cases where interaction between time and condition was found, simple effects were investigated, and Bonferroni correction was used.

Statistical significance was tested at the *p* < 0.05 probability for type I error. *p*-values are presented for two-sided two-independent-sample tests. Statistical analyses were performed with the use of Stata Statistical Software, Release 12 (StataCorp LP: College Station, TX, USA).

## 3. Results

### 3.1. Participants Basic Characteristics

The baseline anthropometric and clinical characteristics of the participants along with their dietary intake and physical activity, as assessed before each arm of the study, are shown in Table 1 and Table 2. The volunteers showed a similar profile before the initiation of each arm. We also compared the baseline anthropometric, biochemical, physical activity, and dietary characteristics of the two sequence groups (PLA/SPI vs. SPI/PLA). No significant differences were observed between baseline measurements for all indices (Supplementary File, Table S1).

### 3.2. Effect of Spirulina Supplementation on Isometric Exercise Performance

**Figure 2:**
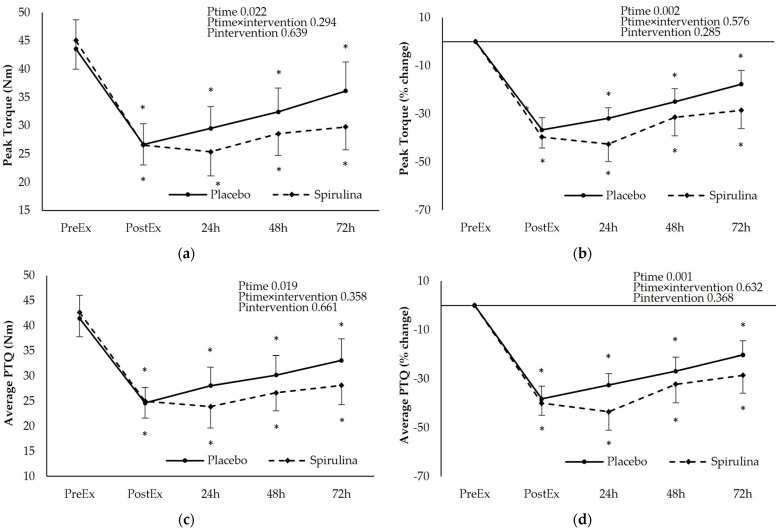
Effect of supplementation on the following: (**a**) peak torque absolute values (Nm); (**b**) peak torque % change; (**c**) average peak torque absolute values (Nm); (**d**) average peak torque % change. * *p* < 0.05 PreEx vs. PostEx, 24, 48, 72 h. Ptime: main effect for time; Ptime × intervention: main effect for time × intervention; Pintervention: main effect for intervention.

The protocol of eccentric exercise induced a sustained decline of Peak Torque (PTQ) and Average Peak Torque (AvPTQ) starting immediately after exercise and lasting until 72 h post-exercise. A post hoc analysis revealed significant time-dependent differences (*p* < 0.05) for both PL and SPI arms. Similar findings were obtained for the percent change of the above-mentioned variables. No significant main effect of “intervention” or “intervention × time” interaction was found indicating that spirulina supplementation was unable to attenuate the reduction of force development capacity of the involved muscle groups. Instead, a non-significant trend for a higher reduction of PTQ and AvPTQ at all time points was observed after the spirulina supplementation compared to the placebo (Figure 2). When the incremental areas under the curve (iAUC) were calculated for PTQ and AvPTQ curves, no significant differences between PL and SPI arms were observed for both PTQ (PL: −45.7 ± 27.4 N.m.h, SPI: −62.4 ± 50.1 N.m.h, *p* = 0.206) and AvPTQ (PL: −45.7 ± 28.0 N.m.h, SPI: −59.7 ± 47.3 N.m.h, *p* = 0.232) 

### 3.3. Effect of Spirulina Supplementation on Delayed-Onset Muscle Soreness (DOMS)

**Figure 3:**
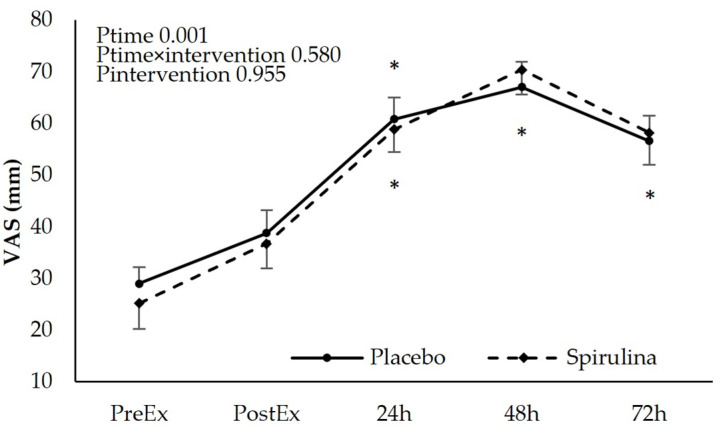
Effect of supplementation on perspective muscle discomfort (Visual Analogue Scale). * *p* < 0.05 PreEx vs. PostEx, 24, 48, 72 h. Ptime: main effect for time; Ptime × intervention: main effect for time × intervention; Pintervention: main effect for intervention.

The subjective perception of discomfort using a “Visual Analogue Scale” increased immediately after the exercise protocol. A post hoc analysis indicated significant time-dependent differences (*p* < 0.05) for both PL and SPI conditions between pre-exercise values and 24 h, 48 h, and 72 h time points. The absolute and % change of soreness perception revealed no significant main effect of “intervention” and “time × intervention” indicating that spirulina supplementation did not affect delayed-onset muscle soreness (Figure 3).

### 3.4. Effect of Spirulina Supplementation on Upper Limb Mobility

**Figure 4:**
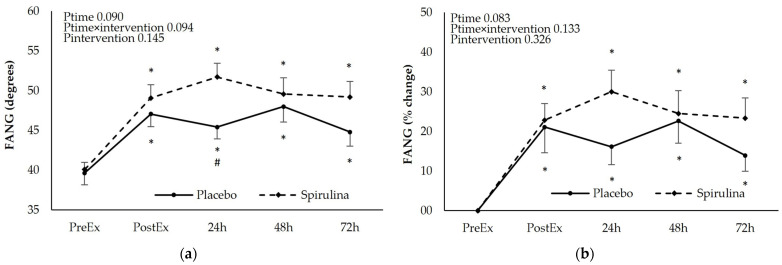

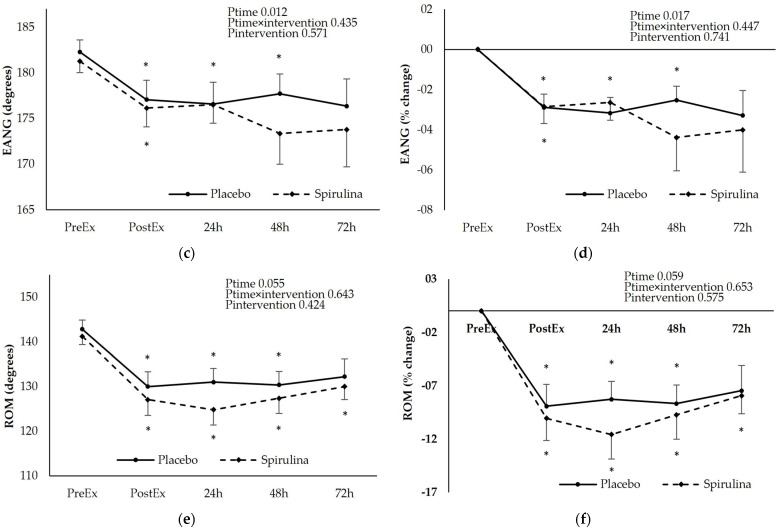
Effect of supplementation on the following: (**a**) flexed angle absolute values (degrees); (**b**) peak torque % change; (**c**) extended elbow absolute values (degrees); (**d**) extended angle % change; (**e**) range of motion absolute values (degrees); (**f**) range of motion % change. * *p* < 0.05 PreEx vs. PostEx, 24, 48, 72 h., # *p* < 0.05 PL vs. SPI for the same time point. Ptime: main effect for time; Ptime × intervention: main effect for time × intervention; Pintervention: main effect for intervention.

For the assessment of upper limb mobility FANG, EANG, and ROM were determined after the eccentric exercise protocol (Figure 4).

The FANG increased immediately after the exercise and remained elevated for 72 h post-exercise (13–22%). Spirulina supplementation did not affect the kinetics of the FANG increase. The EANG changed in an opposite direction showing a small but significant, sustained reduction until 72 h post-exercise. No effect of “intervention” or “time × intervention” was observed although a higher reduction in the EANG was observed at 48 and 72 h post-exercise. Finally, the change of the ROM was similar to that of the EANG showing sustained reduction in both arms of the study (7–10%). Despite the fact that spirulina supplementation did not affect the kinetics of the ROM change, a significantly higher reduction in the ROM was observed in the spirulina arm compared to the placebo 24 h post-exercise. Similar trends were observed when the iAUC between arms were compared for the FANG (PL: 41.7 ± 73.2 deg.h, SPI: 35.0 ± 22.7 deg.h, *p* = 0.766), EANG (PL: −16.5 ± 15.4 deg.h, SPI: −21.2 ± 26.5 deg.h, *p* = 0.509), and ROM (PL: −49.3 ± 34.9 deg.h, SPI: −50.5 ± 34.8 deg.h, *p* = 0.930).

### 3.5. Effect of Spirulina Supplementation on Arm Circumference

**Figure 5:**
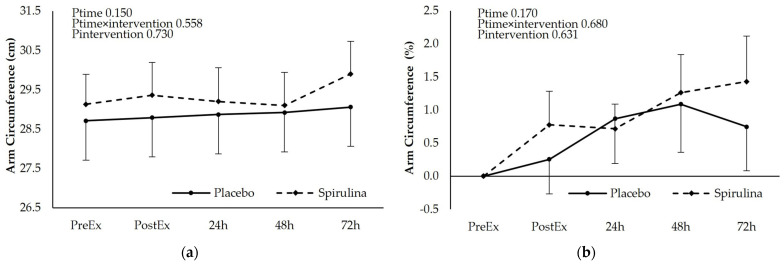
Effect of supplementation on arm circumference as (**a**) absolute values (cm) and (**b**) % change. *p* < 0.05 PreEx vs. PostEx, 24, 48, 72 h. Ptime: main effect for time; Ptime × intervention: main effect for time × intervention; Pintervention: main effect for intervention.

The exercise protocol did not alter arm circumference and no effect of spirulina supplementation was observed (Figure 4). No differences between iAUC were observed (PL: 0.47 ± 1.83 cm × h, SPI: 0.99 ± 1.75 cm × h, *p* = 0.481) (Figure 5).

### 3.6. Effect of Spirulina Supplementation on CK and LDH

A gender-specific effect of exercise on CK and LDH kinetics was observed in our study. Specifically, significant elevations in CK and LDH were observed only for the male subgroup at 48 h and 72 h post-exercise. The exercise protocol could not induce CK and LDH alterations in the female subgroup. Although the time × intervention or intervention effect on CK and LDH was not significant, a trend for higher CK and LDH elevations was observed after the spirulina supplementation (Table 3 and Table 4). The iAUCs of CK and LDH did not differ between groups either for the total population or for each gender, separately. 

## 4. Discussion

Our study investigated the effect of a 2-week *Spirulina Nigrita^®^* supplementation on indices of EIMD in a healthy, moderate physically active population after a standardized eccentric protocol of the non-dominant arm. The specific supplement attracted our attention because of its popularity amongst athletes and physically active people, the non-evidence-based claims on its ergogenic potential, and its ability to enhance recovery. On the other hand, the composition of spirulina which is characterized by a high content of protein, bioactive, antioxidant, and anti-inflammatory compounds such as beta-carotene, vitamin B12, vitamin E, and phycocyanin renders it a promising supplement for the alleviation of EIMD symptoms. However, the outcomes of our study show that *Spirulina Nigrita^®^* supplementation, at doses of 42 mg Kg^−1^ BW per day for 15 days, had no effect on either EIMD symptoms or muscle strength performance after eccentric exercise of the upper non-dominant limb.

The antioxidant and immunomodulating properties of spirulina have been extensively reviewed [15,31,32,33] and its effects are largely attributed to its rich nutrient profile which includes β-carotene, c-phytocyanin, and several vitamins and minerals (recently reviewed in Gogna et al. [15]). Its high concentration of protein and essential amino acids [34,35] could augment availability, and therefore become beneficial for people involved in sports and exercise, in order to reduce post-exercise muscle damage and discomfort, and enhance recovery and muscle tissue regeneration [36,37].

In the present study, we used *Spirulina Nigrita*^®^, a local Greek strain of *Arthrospira plantensis* which has gained extreme popularity as a supplement among the physically active Greek community despite the fact that no dietary intervention studies on an active exercise population have been published so far. Our intention was to investigate the effect of short-term spirulina supplementation on EIMD markers, using a normalized 2-week dosage scheme according to the participant’s body weight. Unlike previously published works [17,19,21,38] in this study we did not use a fixed dose of spirulina but we took into account the differences in body weight between participants. This was of particular importance for the comparability of our results, since our study population included both men and women, with marked differences in body weight. Previous supplementation studies have used fixed doses of 0.5–8 g of different strains of spirulina while the supplementation period ranged from a few days to 8 weeks [39]. In our study, we have chosen a moderate dosage scheme of 42 mg Kg^−1^ BW per day for 15 days which accounts for 2.5–4.2 g of daily consumption of spirulina for participants with a body mass ranging from 60 to 100 kg. The crossover design was chosen to remove inter-participant variation, especially between genders which show significant differences in their responses to eccentric, muscle-damaging protocols. EIMD was induced in the participant’s non-dominant arm as this muscle group is less prone to daily muscle strain and easier to induce EIMD, as opposed to the leg or dominant hand muscle groups, which may be engaged during everyday activity like walking or lifting grocery bags. Finally, we have chosen to recruit young, but moderately physically active participants, without regular participation in training sessions in order to achieve higher EIMD responses since our volunteers were unaccustomed to this kind of exercise. In this way, the putative effect of spirulina supplementation on EIMD markers would be magnified. A six-week washout period has been chosen considering that this duration is long enough to minimize the repeated bout effect and short enough to minimize the dropout rate and changes in dietary intake and physical activity habits. Although, there are reports of a longer-lasting period of repeated bout effect [40] the crossover design of the study should minimize any significant impact of the repeated bout on the ability of spirulina to attenuate EIMD. Table 5 shows that the sequence of supplementation does not alter the iAUCs of EIMD indices after either the placebo or spirulina supplementation. Moreover, it does not alter the delta iAUC (iAUC_SPI_-AUC_PL_) which also implies that the sequence of supplementation did not differentiate the effect of spirulina compared to the placebo. Similar spirulina supplementation studies with a crossover design have shown that even shorter washout periods are adequate to minimize the carry-over effect of spirulina [41].

Anthropometric characteristics and dietary intake remained unaltered before each trial which allowed us to assume that body composition and nutrition did not interfere with the effect of supplementation. The isokinetic eccentric protocol used reduced the power production capacity by 20–37% from the first time point to up to 72 h as evidenced by the post-EIMD decreases of PT and AvPTQ. It also increased perceived discomfort over time while at the same time inducing a sustained lowering of the non-dominant upper limb mobility (ROM). It is therefore obvious that our protocol was able to induce all the typical clinical signs of EIMD although in moderation. Previous studies have shown similar kinetics and magnitude of change after eccentric protocols of the upper limb. Pappas et al. [21], have shown that in a group of 24 healthy recreationally trained males, the eccentric peak torque declined significantly immediately after and up to 72 h post-exercise. Similarly, Chaouachi et al. [38], reported a performance reduction in physical dynamic tests of the lower limbs after three maximal contractions at a single velocity of 30° s^−1^ in elite rugby players.

For all measured indices of EIMD, spirulina supplementation did not alleviate the symptoms of EIMD. Although the statistical analysis did not show a significant time x intervention or intervention effect, a trend for a worsening of the clinical symptoms appeared especially for PT, EANG, and ROM. The effect of spirulina supplementation on eccentric exercise-induced muscle damage was studied in three previous studies. The study of Johnson et al. [41] has shown that the supplementation of *Spirulina platensis* (3 g per day for 8 weeks) had an anti-fatigue effect on healthy male volunteers confirmed by the findings of Lu et al. [17] (7.5 g per day for 3 weeks) and Kalafati et al. [19] where the time to fatigue improved. On the contrary, no effects of spirulina supplementation on EIMD markers after intense exercise were reported by Franka et al. [22] (7.5 g per day), Pappas et al. [21] (6 g per day), and Chaouachi et al. [38], (5.7 g per day for 7 weeks).

Concerning the effect of exercise on the biochemical markers of EIMD, namely CK and LDH, a gender-specific effect was observed since significant elevations of those markers were observed only in the male group at 48 h and 72 h. Additionally, a non-significant trend for higher CK and LDH levels was observed after spirulina supplementation. The majority of our female volunteers were non-responders to CK and LDH. Previous studies that compared the response of these indirect markers of muscle damage between males and females have shown lower elevations of CK and LDH in females than in males [42,43]. This can be attributed to the protective role of estrogens in the breakage of muscle cells, but also to the much lower upper limb muscle mass of females.

It is therefore obvious that our initial hypothesis that *Spirulina Nigrita*^®^ supplementation could alleviate the symptoms of EIMD, is not supported by the results of the statistical analysis. This could be attributed to the dosage scheme (low dose, short duration of the supplementation period). Considering the low bioavailability of phytochemicals, the effective daily dose of *Spirulina Nigrita*^®^ could be much higher. Moreover, the amount of muscle tissue that can be damaged in the upper limbs, is significantly lower compared to the lower limbs. In addition, almost half of the participants were females who had even lower amounts of muscle tissue in their upper limbs and they were resistant to muscle damage. The above may explain the mild effect of our exercise protocol on EIMD indices which in turn did not leave room for spirulina ingredients to exert their actions. It is therefore obvious that we could not rule out that different strains of spirulina or a different dosage may be more efficient on similar exercise protocols.

Our observations were limited by the sample size; possibly, with a larger population, the observed tendencies could reach statistically significant levels. The upper limbs are more prone to EIMD-induced inflammation and oxidative stress, but since upper body EIMD is not common in real life our protocol has less ecological validity than a similar lower limbs eccentric protocol. We used a mixed population of both males and females which showed a different response to CK complicating the assessment of supplementation’s efficiency to alleviate biochemical markers of EIMD. We also used a fixed dosage scheme so we could not draw conclusions on the dose–response effect of spirulina while the 15 days supplementation period may be an insufficient period for spirulina to exert its effects at least at a gene transcription level. Finally, we have not measured redox or inflammation indices which may give a more mechanistic interpretation of our results. On the other hand, the strengths of our study are its randomized, placebo-controlled, crossover design, the usage of *Spirulina Nigrita* ^®^ as a supplement whose efficiency on EIMD has never been investigated before, the assessment of spirulina supplementation on the EIMD of the upper limbs for the first time, the dosage scheme which was controlled by body weight, and the detailed assessment of the dietary intake of our volunteers.

## 5. Conclusions

In conclusion, *Spirulina Nigrita*^®^ supplementation (42 mg Kg^−1^ BW per day for 15 days) did not alleviate symptoms of EIMD or enhance recovery after a damaging eccentric protocol of the upper limbs.

## Figures and Tables

**Table 1:** Basic characteristics of volunteers before each trial.

Basic Characteristics	Placebo	Spirulina	*p*
n	14 (8 males, 6 females)		
Age (years)	24.4 ± 3.9		
BMI (Kg∙m^−2^)	23.0 ± 2.3	23.5 ± 2.5	0.03
Fat (%)	20.8 ± 4.9	21.7 ± 5.6	0.29
WC (cm)	79.6 ± 11	80.9 ± 12	0.18
SBP (mmHg)	115 ± 13	117 ± 22	0.53
DBP (mmHg)	67.0 ± 8.8	69.7 ± 7.1	0.29
HR (bpm)	75.5 ± 14.4	73.8 ± 12.2	0.63
METS (Mets-minute)	1393 ± 1014	1570 ± 1028	0.69

**Table 2:** Dietary intake of macronutrients before each trial.

Dietary Intake	Placebo	Spirulina	*p*
Energy (Kcal)	1945 ± 430	2001 ± 415	0.61
Carbs (%)	44.4 ± 5.8	46.8 ± 7.0	0.25
Protein (g/Kg bw)	1.1 ± 0.28	1.1 ± 0.24	0.58
Protein (%)	15.9 ± 2.7	15.2 ± 2.3	0.23
Fat (%)	38.1 ± 4.2	36.9 ± 6.0	0.54
Fiber (g)	20.5 ± 6.9	23.0 ± 7.8	0.12
Folate (mg)	355 ± 188	356 ± 117	0.69

**Table 3:** Effect of supplementation on creatine kinase activity (absolute values and % change).

CK (IU/L)		PreEx	PostEx	24 h	48 h	72 h	P_time_
Total	PL	96 (54–151) 0.00%	97 (61–183) 3.5% (−5.8–14.1%)	112 (94–242) 9.7% * (−0.54–120.5%)	134 (75–274) 15.8% (−11.9–203.5)	134 * (75–680) 45.8% * (−2.9–322.8%)	0.006
	SPI	86 (66–121) 0.00%	101 (63–135) 20.2% (−9.9–30.3%)	88 (62–272) 6.8% (−4.9–110.9%)	95 * (74–523) 54.8% * (−11.4–424.9%)	101 * (71–1143) 54.9% * (−11.3–551.0%)	0.106
Males	PL	109 (81–211) 0.00%	133 (95–220) 3.46% (−5.8–15.2%)	213 (112–687) 17.89% (−10.9–124.5%)	269 (110–832) 133.9% (−2.5–270.4%)	500 * (106–1002) 260.5%* (−11.5–385.8%)	0.102
	SPI	107 (82–183) 0.00%	120 * (100–226) 24.0% * (15.8–33.4%)	186 (96–564) 89.1% (−3.8–314.2%)	407 (85–1262) 261.4%* (−3.6–644.4%)	708 * (85–1893) 278.7%* (−8.4–1291.8%)	0.308
Females	PL	62 (52–129) 0.00%	61.5 (50–151) 2.7% (−8.9–15.5%)	103 (64–140) 6.7% (0.64–95.7%)	76 (64–145) 2.4% (−24.8–101.0%)	79 (67–145) 8.89% (−20.9–92.7%)	0.621
	SPI	68 (53–86) 0.00%	60.5 (52–88) −9.25% (−28.8–31.2%)	61 (52–74) 5.33% (−19.3–8.4%)	80 (59–106) 37.4% (−25.5–65.6%)	80.0 (55–124) 37.4% (−27.0–88.4%)	0.513

* *p* < 0.05 PreEx vs. PostEx, 24, 48, 72 h.

**Table 4:** Effect of supplementation on LDH activity (absolute values and % change).

LDH (IU/L)		PreEx	PostEx	24 h	48 h	72 h	P_time_
Total	PL	140 (128–178) 0	153 (113–200) 5.2% (−8.1–22.0%)	160 (131–185) 1.1% (−3.3–23.0%)	148 (118–200) 7.7% (−10.6–36.3)	141 (124–189) 6.3% (−7.1–35.6%)	0.84
	SPI	138 (121–188) 0	154 (121–195) 5.9% (−4.6–18.1%)	148 (119–201) –1.9% (−10.8–14.5%)	158 (127–204) 7.5% (–5.8–40.4%)	153 (116–262) 13.6% (−5.9–74.0%)	0.67
Males	PL	154 (132–180) 0%	154 (111–223) 8.1% (−10.7–19.7%)	166 (135–202) 5.1% * (0.87–48.2%)	169 (141–255) 24.6% * (6.8–106.9%)	168 (135–258) 25.5% * (−1.5–95.9%)	0.13
	SPI	139 (122–180) 0%	164 (103–200) 2.2% (−17.9–15.4%)	176 (129–225) 7.9% (−11.2–56.4%)	191 * (158–258) 28.0% * (13.4–99.8%)	215* (140–409) 45,65% * (1.6–130.9%)	0.04
Females	PL	131 (103–161) 0%	153 (119–169) 1.4% (−8.8–25.3%)	157 (118–175) −2.4% (−15.8–16.3%)	118 (108–152) −10.2% (−12.2–5.0%)	132 (100–146) −2.2% (−2.2–13.4%)	0.34
	SPI	134 (117–197) 0%	154 (128–193) 8.1% (−1.9–20.1%)	131 (112–164) −5.6% (−11.3–2.0%)	126 * (117–140) −6.9% * (−22.3–1.5%)	126 (99–165) 2.6% (−27.1–16.0%)	0.045

* *p* < 0.05 PreEx vs. PostEx, 24, 48, 72 h.

**Table 5:** A comparison of iAUCs and delta iAUCs for the EIMD indices according to the sequence of supplementation.

	Placebo–Spirulina Median (25th/75th Percentile)	Spirulina–Placebo	*p*
PT iAUC PL	−62 (−80/−28)	−48 (−58/−10)	0.21
PT iAUC SPI	−39 (−70/−10)	−50 (−125/−30)	0.32
Delta PT iAUC	−6.5 (−22/42)	−27 (−64/−19)	0.053
FANG iAUC PL	25 (6/37)	31 (6/60)	0.62
FANG iAUC SPI	31 (21/33)	37 (25/74)	0.26
Delta FAng iAUC	6.5 (−9/21)	20 (−11/45)	0.53
EANG iAUC PL	−15 (−31/−4)	−9 (−18/−4)	0.62
EANG iAUC SPI	−6 (−22/−1)	−16 (−16.5/−15)	0.10
Delta EANG iAUC	3 (−1.5/25)	−13 (−45/−5.5)	0.01
ROM iAUC PL	−53 (−108/−35)	−56 (−64/−19)	0.16
ROM iAUC SPI	−34 (−53/−27)	−52 (−91/−40)	0.16
Delta ROM iAUC	0 (−5/74)	−25 (−62/−2)	0.038
VAS iAUC PL	100 (61/130)	92 (67/121)	0.71
VAS iAUC SPI	90 (89/149)	95 (70/158)	0.90
Delta VAS iAUC	−8 (−16/60)	23 (−21/90)	0.62
ArmCir iAUC PL	1 (0/2)	−0.15 (−1.55/1.25)	0.32
ArmCir iAUC SPI	0.05 (−0.1/1.4)	2.45 (−0.85/3.3)	0.32
Delta ArmCir iAUC	−0.9 (−1.95/0.6)	1.85 (−2/5)	0.16
CK iAUC PL	232 (−6/370)	25 (−29/747)	0.73
CK iAUC SPI	46 (5/357)	38 (−77/7212)	0.84
Delta CK iAUC	8 (−227/52)	49 (−15/5738)	0.23
LDH iAUC PL	98 (−97/313)	17 (−58/365)	1.00
LDH iAUC SPI	15 (−140/214)	64 (−149/720)	0.71
Delta LDH iAUC	61 (−298/210)	82 (−165/457)	0.71

## Data Availability

The data presented in this study are available from the corresponding author upon reasonable request.

## Supplementary Materials

The following supporting information can be downloaded at: https://www.mdpi.com/article/10.3390/nu16111651/s1, Table S1: Baseline characteristics of volunteers according to supplementation sequence.
