# Supplementary material for: Effect of Spirulina Nigrita® Supplementation on Indices of Exercise-Induced Muscle Damage after Eccentric Protocol of Upper Limbs in Apparently Healthy Volunteers
Source: Nutrients. 2024 May 28;16(11):1651. doi: 10.3390/nu16111651 (PMC11174877; doi:10.3390/nu16111651)
Supplement: Supplementary file 1 [file nutrients-16-01651-s001.zip › nutrients-2938472-supplementary.pdf]

Table S1. Baseline characteristics of volunteers according to supplementation sequence

**Sequence**	**Placebo-Spirulina**			**Spirulina-Placebo**		
	Placebo	Spirulina	**p[1]**	Placebo	Spirulina	**p[2]**
BMI (Kg·m^{-2})	23.2 ± 2.7	23.8 ± 2.9	0.062	22.7 ± 1.9	23.1 ± 2.3	0.310
Fat (%)	21.7 ± 6.1	23.4 ± 6.4	0.154	19.9 ± 3.6	19.9 ± 4.6	0.972
SBP (mmHg)	114 ± 17	109 ± 10	0.278	118 ± 22	123 ± 25	0.102
DBP (mmHg)	67 ± 8	64 ± 4	0.433	67 ± 9	74 ± 7	0.061
HR (bpm)	72 ± 16	70 ± 8	0.622	79 ± 12	77 ± 15	0.669
Energy (Kcal)	1967 ± 473	1944 ± 468	0.875	1924 ± 420	2058 ± 383	0.471
Carbs (%)	43 ± 3.6	46 ± 5.7	0.419	46 ± 7.6	48 ± 8.4	0.464
Protein (%)	17 ± 2.8	15 ± 2.7	0.114	15 ± 2.4	15 ± 2.1	0.831
Fiber (g)	20 ± 7.3	24 ± 6.1	0.285	21 ± 7.1	22 ± 9.7	0.268

[1] p values for paired samples t-test for the comparison of pre-exercise values in PL and SPI arms (sequence PL → SPI)

[2] p values for paired samples t-test for the comparison of pre-exercise values in PL and SPI arms (sequence SPI → PL)
